# Examining the applicability of virtual battle space for stress management training in military personnel—A validation study

**DOI:** 10.1002/smi.3475

**Published:** 2024-09-28

**Authors:** Frank P. M. Schilder, Frederique M. Withagen, Antoin D. de Weijer, Bastiaan Bruinsma, Elbert Geuze

**Affiliations:** ^1^ Department of Psychiatry University Medical Centre Utrecht The Netherlands; ^2^ Brain Research and Innovation Centre Ministry of Defence Utrecht The Netherlands; ^3^ University of Utrecht Utrecht The Netherlands

**Keywords:** cognitive performance, simulator, stress response, validation study, virtual battle space

## Abstract

Military personnel are often exposed to high levels of both physical and psychological challenges in their work environment and therefore it is important to be trained on how to handle stressful situations. The primary aim of this study was to examine whether military‐specific virtual battle space (VBS) scenarios could elicit a physiological and subjective stress response in healthy military personnel, as compared to that of a virtual reality height exposure (VR‐HE) stress task that has shown to reliably increase stress levels. Twenty participants engaged in two VBS scenarios and the VR‐HE during separate sessions, while measurements of heart rate (HR), heart rate variability (HRV), respiration rate, and subjective stress levels were collected. Contrary to our initial expectations, analysis revealed that neither of the VBS scenarios induced a significant stress response, as indicated by stable HR, HRV, and low subjective stress levels. However, the VR‐HE task did elicit a significant physiological stress response, evidenced by increased HR and HRV changes, aligning with previous research findings. Moreover, no discernible alterations were detected in cognitive performance subsequent to these stressors. These results suggest that the current VBS scenarios, despite their potential, may not be effective for stress‐related training activities within military settings. The absence of a significant stress response in the VBS conditions points to the need for more immersive and engaging scenarios. By integrating interactive and demanding elements, as well as physical feedback systems and real‐time communication, VBS training might better mimic real‐world stressors and improve stress resilience in military personnel. The findings of this study have broader implications for stress research and training, suggesting the need for scenario design improvements in virtual training environments to effectively induce stress and improve stress management across various high‐stress professions.

## 1 INTRODUCTION

Military personnel are often exposed to high levels of both physical and psychological challenges in their work environment (Harris et al., 2005). It is therefore important for military personnel to be trained how to cope with stressful situations. In recent years, simulation software has become an important tool for military training improvement (Curry et al., 2016; Whitney et al., 2014), yet simulation software is not frequently used to teach their personnel how to cope with stressful events. This study aims to bridge that gap by testing the use of simulation software for inducing stress to aid in military training sessions.

Stress that is caused by the work environment of military personnel can have a negative impact on cognitive functions, like working memory, attention, cognitive flexibility, and inhibition (Lieberman et al., 2016; Shields et al., 2016). The extent to which these negative effects appear depends on the amount of stress. This relationship is often described as an inverted U‐shape curve (Sapolsky, 2015). More specific, at moderate levels of stress, individuals tend to experience heightened alertness and focus, which can lead to improved cognitive performance (Shields et al., 2019). This optimal level of stress is often referred to as ‘eustress’ and can enhance problem solving abilities and memory functions. However, as stress exceeds beyond this optimum level, cognitive performance can decline. For example, high levels of stress can lead to anxiety symptoms (Grillon et al., 2007), impaired concentration (Eskildsen et al., 2015), and memory deficits (Guenzel et al., 2013). In short, the relationship between stress and cognitive performance resembles a curve in which both extremely low and extremely high levels of stress can have negative effects, while a moderate level of stress can enhance cognitive performance.

In military context, it is therefore important to minimize the negative effects of stress on the cognitive capacity of military personnel. Namely, it is known that a decline in cognitive functioning is related to battlefield errors (e.g., friendly fire incidents, collateral damage) (Morgan et al., 2006). To minimize these negative effects, an extensive military training curriculum is used to train military personnel to cope with different stressful situations, exposing them to unpredictable and uncertain circumstances. These trainings have been developed to induce both physical and psychological stress (Vartanian et al., 2018). In the last decade, virtual training applications to develop military skills, such as communication, tactics, and shooting, are predominantly used (Curry et al., 2016). These virtual training tools have made mission‐oriented training more efficient and can be used as mission preparation training. Moreover, simulation software may help make mission‐oriented training more affordable and practical seeing that it enables creation of a vast array of scenarios with little to no supplies needed (Michael et al., 2022). Besides, it may be a great tool for research purposes, as it could help making laboratory paradigms for investigating the stress response more controllable and realistic (Tarr & Warren, 2002).

In recent years, virtual reality (VR) has emerged as a powerful tool in scientific research and practical applications. The immersive environments offer unique opportunities to recreate realistic scenarios and open the possibility of developing novel stressors that could not be recreated easily in real‐life environments (Dammen et al., 2022). For example, virtual height exposure demonstrated an increased stress response displayed by physiological measurements (e.g. Heart rate (HR), HR variability or electrodermal activity) (Kisker et al., 2021; Martens et al., 2019; Peterson et al., 2018; Zhu et al., 2023). Moreover, VR scenarios that replicated military combat (Bouchard et al., 2014; Linssen et al., 2022), medical emergencies (Chang et al., 2021; Ghazali et al., 2016), or trauma and phobia exposure (van ’t Wout et al., 2017) also showed an increase in stress levels. Consequently, VR provides a controllable environment for studying behaviour that is ideally suited for stress management training. For example, it allows to vary in interactive training scenarios, either by system's AI or by a human operator. In addition, it facilitates behavioural conditioning by providing instant feedback when individuals employ stress management techniques. Moreover, repeated exposure can potentially help desensitize individuals to the exposed stressors (Grissom & Bhatnagar, 2009; Kothgassner et al., 2021; Pallavicini et al., 2016).

Despite the promising potential of virtual training for stress management, its application in military populations has been rare. VBS provides a whole‐earth virtual desktop training environment that provides creation and simulation of any imaginable military training scenario. In the Dutch military, VBS is mainly used for communication and navigation training. Despite its broad scope, it is not yet being used for specific stress‐training purposes. So, there is a need for a realistic simulation that can mimic stressful situations, allowing trainees to learn and develop coping strategies for real‐world scenarios. For example, VBS can be used to create a vast array of scenarios with different types of stressors and events that resemble mission details and environments that one could expect during the actual mission, as done by Binsch et al. (2021). Here, they developed a passive patrol scenario that added stressors, such as task difficulty, auditory noise, and electric muscle stimulation in a stepwise manner over different phases (Binsch et al., 2021). Their study, however, did not yield significant results utilising VBS to induce a stress response. However, by building upon the work of Binsch et al. and integrating more diverse, interactive and demanding goal‐directed scenarios within the VBS framework, our study aims to determine if such adjustments can indeed evoke measurable changes in individual stress levels.

Therefore, we will compare the stress response of VBS to that of the virtual reality height exposure (VR‐HE) task, which has demonstrated an increase in stress parameters reliably (Kisker et al., 2021; Martens et al., 2019; Peterson et al., 2018). We hypothesize that both VBS and the VR‐HE demonstrate an increase in stress parameters. Taking into consideration the findings of Lieberman et al. (2016) and Shields et al. (2016), our second aim is to examine the effect of a virtually induced stress response on cognitive functioning. We hypothesise that, as both tools are expected to induce a stress response, cognitive performance will decrease in both conditions.

## 2 METHODS

This study was preregistered at OSF (www.osf.io, STRIVE) and was approved by the faculty ethical committee of Utrecht University (FETC).

### 2.1 Participants

Twenty servicemembers (16 males/four females) participated in this study and were recruited from various military bases of the Dutch Ministry of Defence. Military ranks were different amongst participants, and included soldiers (7), corporals (9), sergeants (2), and lieutenants (2). Participants were included within an age range between 18 and 40 years old. The mean age of the participants was 26.5 (SD = 5.24) (see Table 1). Those with physical health issues or a diagnosis of a psychiatric disorder (except for ADHD and autism) were excluded. Additionally, participants who had a diagnosis of an endocrine disorder or sleep disorder were excluded as well. Exclusion criteria were established beforehand by a telephone screening procedure. Participants were instructed not to drink any alcohol the evening before the experiment and not to drink coffee or smoke at least 2 hours before the experiment. In an a‐priori power calculation using the difference between two dependent means (before and after the stressor), we calculated that in order to obtain a power of 0.80 with an alpha level of 0.05 (two‐sided) and using the effect size of 0.68 that was derived from stressful VR exposure on HR (Dammen et al., 2022), 19 participants were needed (G*Power, v3.1). Post hoc power analysis demonstrated that with a Cohen's F of 1.38 (main effect of time on HR), a power of 1.0 was achieved. All participants provided written informed consent. The authors assert that all procedures contributing to this work comply with the ethical standards of the relevant national and institutional committees on human experimentation and with the Helsinki Declaration of 1975, as revised in 2008.

**TABLE 1:** Demographic characteristics of the study population and videogame experiences.

Demographics	*N* (%)
Age	20
Mean (SD)	26.5 (5.24)
Sex	
Male	16 (80)
Female	4 (20)
Highest education level	
High school	3 (15)
MBO (vocational education)	15 (75)
HBO (higher professional education)	1 (5)
WO Bachelor (university)	1 (5)
Military branch	
Royal land force	16 (80)
Royal air force	1 (5)
Royal navy	2 (10)
Royal military police	1 (5)
Military rank	
Enlisted	16 (80)
Non‐commissioned officer	2 (10)
Commissioned officer	2 (10)
Years of employment	
1–3 years	6 (30)
3–6 years	9 (45)
6–10 years	3 (15)
10+ years	
Experience with VBS	
No experience	16 (80)
Playing videogames	
Never	5 (25)
Sometimes	7 (35)
Often	4 (20)
Very often	4 (20)
Hours playing videogames per week	
0 h	5 (25)
1–2 h	7 (35)
3–5 h	3 (15)
5–6 h	2 (10)
6+ hours	3 (15)

Abbreviation: VBS, virtual battle space.

### 2.2 Design

**FIGURE 1:**
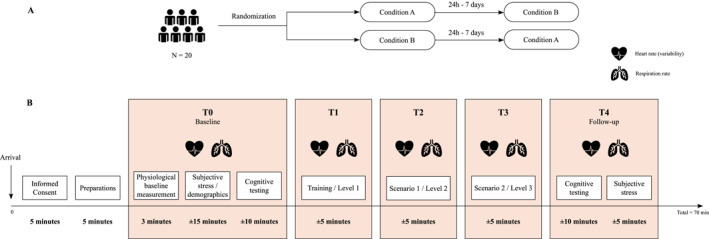
A schematic overview of the study procedure. Before the start of the study the participant is (a) randomised into either Condition A or B and completes both conditions on separate visits. (b) Visualisation of the first session and measurements. HR and HRV measurements were taken throughout the entire experiment session. Saliva samples were taken after each timepoint. HR, heart rate; HRV, heart rate variability.

In a within‐subject design, military subjects underwent two different experimental conditions, each investigating the effect on stress levels using a stress task, namely the VR‐HE and the VBS task, on separate visits. The order of the tasks was randomised. Both conditions incorporated three phases with a rest phase before the experimental task (baseline). During the initial rest phase, we established baseline stress levels. This was immediately followed by cognitive testing. The VBS condition consists of three different scenarios: a tutorial and two stress‐inducing scenarios. The VR‐HE condition consists of three VR levels. After both conditions, a follow‐up is done to, again, establish stress and cognition levels. An overview of the study design is demonstrated in Figure 1.

### 2.3 Materials

For this study, we used military simulator software VBS (Bohemia Interactive^®^) version 4.22.2 to develop and simulate two desktop scenarios. Scenarios were displayed on a large computer monitor (LG 34UC79 G; 2560x1080), and a keyboard and mouse were used for simulation controls.

For the VR‐HE simulation, the participants were equipped with the HTC Vive Pro 2 with Richie's Plank Experience, a VR height simulator. The HTC Vive Pro controllers were used to interact with the VR environment (pushing buttons and picking up objects). To increase immersion in the VR environment, a real‐world wooden plank (3.0 m × 0.2 m) was placed on the floor to walk on and a table fan simulated a headwind whenever the participant was on the plank. Both simulations ran on the MSI GE76 Raider.

The BIOPAC MP160 was used for physiological data acquisition, electrocardiogram (ECG) and respiration. Disposable electrodes (EL504) on the torso were also acquired from BIOPAC© and were used to assess ECG data. Respiratory data was obtained with the BioNomadix Respiration Transducer (BN‐RESP‐XDCR). Physiological data was obtained with a laptop running the AcqKnowledge^®^ software (BIOPAC©).

### 2.4 Procedure

This study was conducted on multiple military bases in The Netherlands. All participants were tested individually. The complete experiment consisted of two separate visits that lasted about 70 min per participant for the first visit, and 50 min for the second. Visits typically occurred with a one‐day interval, and at most, within a week of each other. Data was collected during weekdays between 9 AM and 5 PM, with each participant's data collected at the same time of day for both visits.

#### 2.4.1 Preparations and data collection

Before the first session, participants were randomised into the VBS or VR‐HE condition using block randomisation. After welcoming the participant, procedures were explained and informed consent was signed. Thereafter, the BIOPAC system was connected. For ECG measurements, electrodes were placed 1 cm under the collarbone on the right and the left side. A third electrode was placed on the left side (participants view), in a direct line with the other electrode, between the last two ribs. The ECG data was sampled using a rate of 1000 Hz and a 0.5–35 Hz ECG filter. Additionally, a single respiratory band was placed around the waist at the height of the sternum. Respiratory data was sampled at 61.5 Hz.

When installed, the baseline measurement (T0) was started to determine a baseline level of physiological stress parameters. In the VBS condition, baseline values were determined by instructing the participant to sit still and relax for 3 min while fixating on a cross that was displayed on a monitor. To account for the movement in the VR‐HE condition, participants were instructed to walk leisurely around the room for 2 min. The baseline value was then determined by sitting still for 1 min to account for a possible shift in ECG parameters. Next, the two questionnaires (see below) were administered to assess the level of subjective stress. Subsequently, the participants performed the cognitive tasks to establish baseline cognitive performance. After the stressor (T4), the cognitive tasks were administered again to measure post‐stress cognitive performance. Subsequently, the questionnaires were completed again. The physiological stress parameters (HR, HRV and respiration) were measured throughout the entire experiment.

### 2.5 Stress conditions

#### 2.5.1 Virtual Battle Space

The simulation consisted of three parts and began with an introductory training scenario (T1). In this scenario we simulated a training ground to learn the controls of VBS and practice the most basic actions such as walking, running, crawling, and shooting. The duration of the training varied and depended on how fast the participant completed the challenges. Also, the training scenario contained no stress‐inducing elements, and its sole purpose was to get acquainted with the software. The training scenario was succeeded by two scenarios that simulated challenging environments, in which participants were engaged in strategic thinking and foreseeing unfolding events. Both scenarios adhered to a similar structure comprising an observation phase and an ambush phase, trying to elicit a stress response by employing time restraint, threat, uncertainty, uncontrollability, and distraction. All encountered situations have been part of the basic military training curriculum and should be familiar to all participants.

During the first scenario (T2), participants were assigned to guard a bridge. After a few minutes an explosion occurs, and the participant is ambushed by two shooters. During the ambush a truck drives up to the bridge and explodes. In the second scenario (T3), the participant is ambushed at nightfall by a larger group of enemies. The participant is tasked with guiding the injured colleagues to safety.

#### 2.5.2 The virtual reality height exposure task

The VR simulation, the VR‐HE, also comprised three distinct phases. However, unlike VBS, there was no training phase. Instead, we progressively introduced additional stress‐inducing aspects every phase, aiming to increase the stress levels. The VR‐HE task utilised a natural fear of heights (acrophobia) and fear of falling as its main tactics to elicit a stress response. The entirety of the simulation took roughly 15 min to complete.

Initiating the simulation, participants ascended a virtual elevator to the top floor of a skyscraper, simulating an altitude of approximately 160 m. In the first phase (T1), participants were tasked with traversing a plank and, upon reaching its edge, verbally identifying the number of windmills visible in the distant landscape. Subsequently, they were instructed to rotate clockwise and return to the elevator. Descending to ground level, they awaited a brief pause (approximately 30 s) before commencing the next level.

The second phase (T2) was similar to the first, with the addition of objects placed at the plank's end. Participants were directed to advance towards these objects, pick them up (specifically, doughnuts on a plate), and consume them (by moving the controller towards their mouth) before repeating the process until all objects were acquired. Upon completion, they turned around and returned to the ground floor.

For the third phase (T3) the participants were instructed to press the number ‘6‐6‐6’ in the elevator. This started the built‐in horror level of the software. This phase unfolded with a gradual approach of a large spider towards the open elevator, resulting in the spider leaping towards the participant. After, the elevator would take the player up to the plank where a phone was ringing at the end. Upon answering the phone, the plank disappeared, and the player fell towards the ground. The phase then transitioned to a dark room where a dentist wielding a drill advanced towards the player. The sequence concluded with the participants finding themselves in the middle of the street, where a bus collision would abruptly terminate the simulation if the participant would turn around.

#### 2.5.3 Validation experiment

Data examination revealed changes in cardiac parameters after standing up in the VR‐HE condition. However, because this was not anticipated, data could not be analysed reliably in the current dataset. Moreover, the 2‐min walking phase as a baseline had a greater effect on cardiac measurement compared to the movement during the VR task. Therefore, we conducted a separate experiment among colleagues to replicate the phenomenon. Nine colleagues were asked to sit still for 3 min and then immediately stand up and stand as still as possible for another 3 min. HR and HRV data were collected using the BIOPAC MP160.

### 2.6 Dependent measures

#### 2.6.1 Questionnaires

The assessment of subjective stress levels involved the utilisation of the Dutch version of the State‐Trait Anxiety Inventory (STAI) developed by H.M. Van der Ploeg (1980). Comprised of 40 items, this inventory evaluates both state and trait anxiety, respectively. Each item is rated on a 4‐point scale ranging from ‘almost never’ to ‘almost always’ with higher scores indicating greater anxiety. Notably, scores pertaining to items reflecting positive feelings were reversed. Furthermore, the Dutch version of the visual analogue scale (VAS) (Hayes & Patterson, 1921; Hornblow & Kidson, 1976) was utilised to evaluate subjective stress. This scale prompted participants to indicate their level of tension on a scale of 1–10, with 1 representing no tension and 10 indicating maximum tension. Both the STAI and the VAS were administered both before and after each stress‐inducing condition (T0 and T4) to capture changes in subjective stress levels.

#### 2.6.2 Cognitive functioning

To evaluate the impact of stress on cognitive functioning, participants underwent two cognitive tasks: the Stroop task and the psychomotor vigilance task (PVT). Both tasks were administered both before and after each stress condition (T0 and T4), aiming to capture potential changes in cognitive performance. The development and administration of these cognitive tasks were facilitated through Psychopy version 2021.1.1.4 (Peirce, 2007). Task order was not randomised.

The Stroop task, designed to assess selective attention and processing speed (MacLeod, 1992), involved presenting words in various colours. The colour of the letters could be either congruent (matching the word's colour) or incongruent (not matching the word's colour). Participants were required to name the colour of the letters (the ‘ink’) by pressing a corresponding key on the keyboard. The outcome measure was the reaction time on incongruent trials minus the reaction time on congruent trials, referred to as the ‘Stroop effect’. The task consisted of 72 trials and took approximately three minutes to complete.

Subsequently, the PVT was employed to gauge vigilance, denoting a focused attention state facilitating the identification of specific occurrences during periods of low activity (Wilson et al., 2010). In this task, participants were tasked with pressing the ‘spacebar’ on the keyboard when the stimulus colour changed from white to red, aiming to react as promptly as possible. The outcome measure of the PVT was reaction time, and the task required 5 min to complete.

### 2.7 Data analysis

The BIOPAC AcqKnowledge software was employed for the analysis of the physiological data, specifically focussing on HR, HRV and respiration rate (RR). A baseline measurement (T0) was conducted to establish a reference point for subsequent comparisons. In the VR‐HE condition, despite required movement during the task, we decided to neither correct for walking effects nor the effect of standing up. This choice was, first, based on the negligible impact that walking during the VR task had on the ECG data and, second, the fact that individual differences in ECG measurements after standing up could not be analysed reliably in the current dataset.

#### 2.7.1 Heart rate (variability)

For the HRV assessment, a time domain analysis, specifically Root Mean Square of Successive Differences (RMSSD) was executed. This analysis relies on the intervals between consecutive heartbeats. The RMSSD, or the RMSSD between a set of heartbeats (Pham et al., 2021), was computed using the R‐R interval, representing the time between two heartbeats.

$$\text{RMSSD}=\sqrt{\frac{\sum_{i=1}^{N-1}{\left({{\text{RR}}_{i}-\text{RR}}_{i+1}\right)}^{2}}{N-1}}$$

The resulting RMSSD score serves as an indicator of autonomic nervous system (ANS) activity, with a higher score correlating with parasympathetic activity and a lower score reflecting sympathetic activity (Hoareau et al., 2021). RMSSD scores were computed over 30‐s intervals. Similar to RMSSD time, intervals between adjacent R peaks (R‐R intervals) from the QRS complex were used to calculate the mean HR in beats per minute for every timepoint.

#### 2.7.2 Respiration rate

Prior to the analysis of the respiration data, a pre‐processing step involved transforming the entire waveform to a frequency of 62.5 Hz. Subsequently, a band‐pass filter was applied, using fixed values of 0.05 for the low‐frequency component and 1.0 for the high‐frequency component. Following these pre‐processing steps, the mean RR was calculated for every timepoint. Ultimately, only data at T0 and T4 could be presented due to exclusion of unreliable data during movement at T1 to T3 in the VR‐HE condition.

### 2.8 Statistical analysis

All data was analysed using IBM SPSS Statistics (Version 29.0.1.1).

#### 2.8.1 Stress

To analyse the difference in both HR and RR between the VBS and the VR‐HE condition across the different time points, a two‐way repeated measures ANOVA was used. Similarly, for the analysis of the difference in HRV between the VBS and the VR‐HE condition over the different time points, a two‐way repeated measures ANOVA was used. Finally, to explore the difference in subjective stress between the VBS and the VR‐HE condition over the different time points, a two‐way repeated measures ANOVA was used as well.

#### 2.8.2 Cognition

To analyse the difference in cognitive performance between the VBS and the VR‐HE condition over the different time points, a two‐way repeated measures ANOVA was used.

#### 2.8.3 Validation experiment

To analyse the difference between sitting and standing a paired‐samples *t*‐test was used for HR, and a related‐samples Wilcoxon Signed Rank Test was used for HRV, because HRV data was not normally distributed.

#### 2.8.4 Assumptions

The Shapiro‐Wilk test for normality was performed to see whether the data was normally distributed and revealed three out of the 16 variables were not. Despite these three not normally distributed variables, the two‐way repeated measures ANOVA can still be performed, since the *F*‐test of an ANOVA is robust for non‐normality (Blanca et al., 2017). A Mauchly's Test of Sphericity revealed that sphericity was violated in multiple ANOVAs. To correct for this violation, a Greenhouse‐Geisser correction was used when interpreting the results of these ANOVAs.

#### 2.8.5 Corrections

In order to address the issue of multiple comparisons, we applied the Benjamini‐Hochberg procedure for False Discovery Rate correction to all *p*‐values obtained from our analyses (Benjamini & Hochberg, 1995). This correction offers a powerful means of controlling for Type I errors. The corrected *p*‐values are presented alongside the original *p*‐values in Table S2. When the ANOVAs revealed a significant *F* test, post‐hoc tests were conducted incorporating a Bonferroni adjustment.

## 3 RESULTS

### 3.1 Participants

A total of 20 participants successfully completed the study. However, the analysis of HR and HRV excluded two participants owing to corrupted ECG data, resulting in an effective sample size of 18 participants. In contrast, for respiration, subjective stress, and cognition data, no participants were excluded, leading to an intact dataset encompassing all 20 participants.

### 3.2 Physiological measures

#### 3.2.1 Heart rate

**FIGURE 2:**
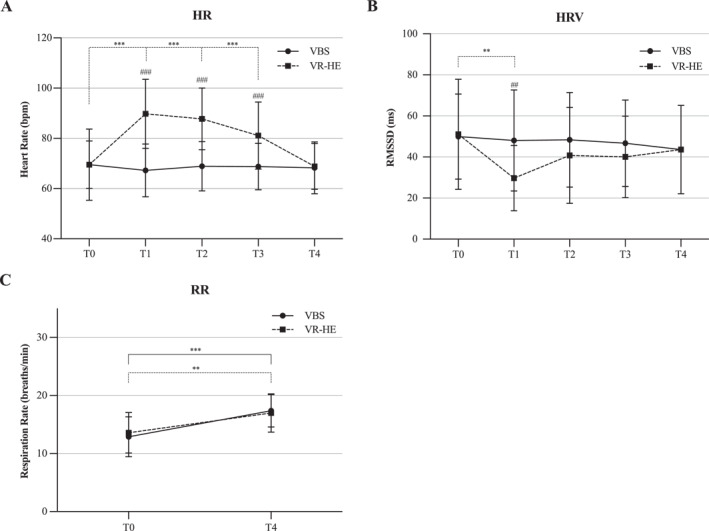
An overview of the change in physiological stress in both stress conditions across the experimental time course shown as mean ± SD. HR, heart rate; HRV, heart rate variability; RR, respiration rate; VBS, virtual battle space; VR‐HE, virtual reality height exposure. *p < 0.05; **p < 0.01; ***p < 0.001 (within‐group); #*p* < 0.05; ##*p* < 0.01; ###*p* < 0.001 (between‐group).

The 5 (Time) × 2 (Condition) ANOVA on HR showed a significant main effect of time, F(2.678) = 32.465, *p* < 0.001; a significant effect of stress condition, F(1) = 32.606, *p* < 0.001; and a significant interaction effect, F(2.565) = 53.368, *p* < 0.001. Post‐hoc comparisons indicated a significant increase in HR in the VR‐HE condition at T1 (*p* < 0.001), T2 (*p* < 0.001) and T3 (*p* < 0.001) when compared to baseline. Contrarily, no significant differences in HR were observed in the VBS condition, F(4) = 2.042, *p* = 0.098. Consequently, HR was significantly higher at T1, T2 and T3 in the VR‐HE condition compared to VBS (*p* < 0.001). These outcomes are visually represented in Figure 2 and an overview of the averaged outcomes is presented in Table S1.

#### 3.2.2 Heart rate variability

The 5 (Time) × 2 (Condition) ANOVA on HRV revealed a significant main effect of time, F(4) = 5.059, *p* = 0.0042; no significant effect of stress condition (*p* = 0.113); and a significant interaction effect, F(4) = 4.330, *p* = 0.014. Post‐hoc comparisons showed a significant decrease of RMSSD in the VR‐HE condition only at T1 (*p* < 0.001) compared to baseline, and when compared to the VBS condition (*p* = 0.003). No differences were observed in the VBS condition, F(4) = 0.678, *p* = 0.610. HRV outcomes are presented in Figure 2 and an overview of the averaged outcomes is presented in Table S1.

#### 3.2.3 Respiration rate

The 2 (Time) × 2 (Condition) ANOVA on RR showed a significant main effect of time, F(1) = 35.127, *p* < 0.001; no significant main effect of stress condition (*p* = 0.870); and no significant interaction effect (*p* = 0.180). Post‐hoc comparisons demonstrated that RR was significantly higher at the end of the experiment in both the VR‐HE (*p* = 0.005) and the VBS condition (*p* < 0.001). Outcomes are presented in Figure 2.

#### 3.2.4 Subjective stress

**FIGURE 3:**
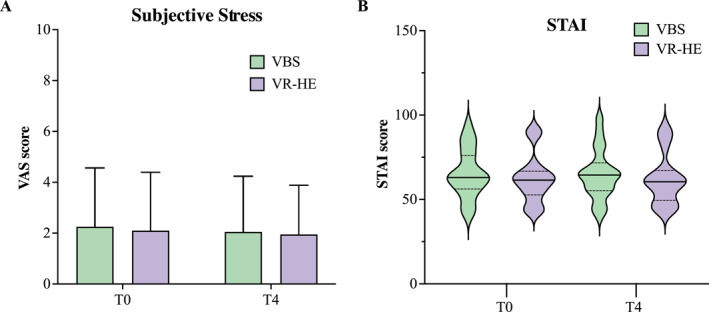
An overview of the change in subjective stress in both stress conditions across the experimental time course shown as mean ± SD. VAS, visual analogue scale; VBS, virtual battle space; STAI, state‐trait anxiety inventory; VRHE, virtual reality height exposure.

The 2 (Time) × 2 (Condition) ANOVA on the VAS and STAI scores showed no significant main effects of time, condition, nor interaction. Outcomes are presented in Figure 3 and an overview of the averaged outcomes is displayed in Table S1.

### 3.3 Cognition

#### 3.3.1 Psychomotor vigilance task

**FIGURE 4:**
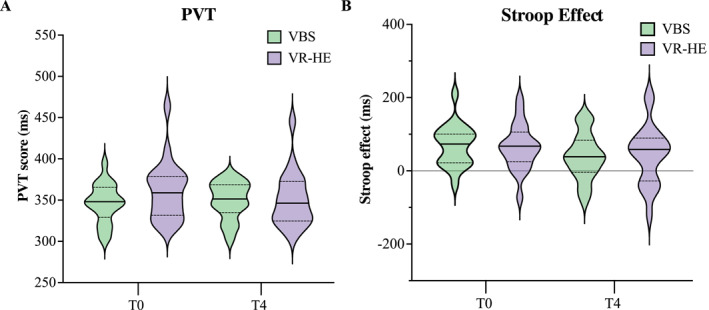
An overview of the change in PVT and Stroop scores in both stress conditions across the experimental time course shown as mean ± SD. PVT, psychomotor vigilance task; VBS, virtual battle space; VR‐HE, virtual reality height exposure.

The 2 (Time) × 2 (Condition) ANOVA on PVT scores showed no significant main effects of time and stress condition, nor interaction. PVT outcomes are presented in Figure 4 and an overview of the averaged outcomes is displayed in Table S1.

#### 3.3.2 Stroop task

The 2 (Time) × 2 (Condition) ANOVA on the Stroop effect showed no significant main effects of time and stress condition, nor interaction. Stroop outcomes are presented in Figure 4 and an overview of the averaged outcomes is presented in Table S1.

#### 3.3.3 Validation experiment

**FIGURE 5:**
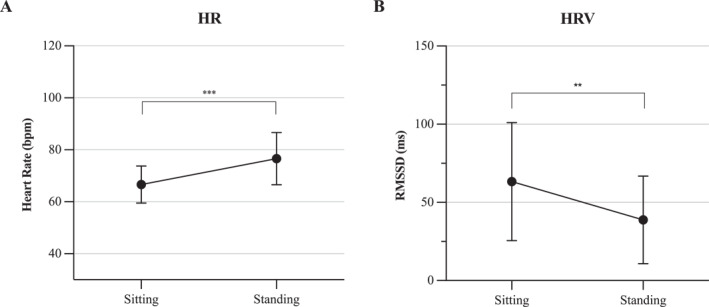
An overview of the change in ECG parameters after standing up shown as mean ± SD. Data was collected from nine colleagues. HR, heart rate; HRV, heart rate variability. *p < 0.05; **p < 0.01; ***p < 0.001.

A paired‐samples *t*‐test was conducted to compare HR values between sitting and standing. The test revealed a significant increase in HR after standing‐up (*t*(8) = −6.610, *p* < 0.001). For HRV, the Wilcoxon Signed Rank Test was conducted and revealed a significant decrease in RMSSD (*Z* = 0.000, *p* = 0.008). This indicates that ECG parameters change after standing up, and that this effect remained for up to 3 min. Outcomes are presented in Figure 5.

## 4 DISCUSSION

The primary aim of this study was to examine whether military‐specific VBS scenarios could elicit a physiological and subjective stress response in healthy military personnel, as compared to a previously validated VR stress task (VR‐HE). Participants engaged in two VBS scenarios and the VR‐HE during separate sessions, while measurements of HR, HRV, RR, and subjective stress levels were collected. Our hypothesis posited that stress levels during both conditions would be higher during the task than at baseline (T0) and follow‐up (T4). The second objective aimed to explore the impact of both tasks on cognitive functioning. Here, we anticipated a discernible impairment in cognition following a measurable stress response in both conditions.

Contrary to our initial expectations, analysis of HR and HRV data revealed that neither of the VBS scenarios induced a significant stress response. Similarly, overall subjective stress levels were low and remained unaltered following VBS exposure. However, the VR‐HE condition did result in increased HR, indicating higher stress levels during the task. These findings align with previous studies utilising versions of the VR‐HE (Kisker et al., 2021; Martens et al., 2019; Peterson et al., 2018; Zhu et al., 2023). Notably, HR increased significantly at level 1 (T1) from baseline, a trend that persisted through to level three (T3). The consistently higher HR across all VR‐HE levels compared to VBS scenarios suggests that participants experienced more stress during the VR‐HE.

In general, RMSSD values revealed a decline from baseline to T1, and more specifically, this decrease was significantly lower compared to the VBS condition. However, no discernible difference in HRV was observed between the VBS and VR‐HE conditions at the subsequent three timepoints. As previously discussed, this outcome aligns with expectations, considering the non‐stress‐inducing nature of the training scenario. Interestingly, contrary to our observations in HR, these results do not lend support to the notion that VR‐HE levels were more stressful than the VBS scenarios.

The observed increase in HR following the VR‐HE task, coupled with stable HRV, warrants a deeper exploration. One potential explanation is the distinction between sympathetic (SNS) and parasympathetic nervous system activities. Namely, HR is primarily influenced by the ANS, specifically the balance between sympathetic and parasympathetic activities (McCorry, 2007). Therefore, an increase in HR typically indicates heightened sympathetic activity, suggesting a stress response. However, HRV, particularly RMSSD, reflects the overall balance and interaction between the SNS and ANS over time (Gullett et al., 2023). The discrepancy between HR and HRV might therefore be due to predomination of the sympathetic nervous system causing an increase in HR. Yet, HRV reflects short‐term variations in heartbeats and is primarily driven by parasympathetic activity (Gullett et al., 2023), and may not decrease correspondingly if the parasympathetic withdrawal is not pronounced or if there is a rapid compensatory recovery. In other words, the VR‐HE task may have induced a state of vigilance/arousal or cognitive engagement (Canisius & Penzel, 2007; Jerčić et al., 2020) that elevates HR without causing significant mental stress, which would more markedly affect HRV (Boonnithi & Phongsuphap, 2011). Additionally, military personnel might have a more resilient autonomic response, allowing them to maintain relatively stable HRV even when HR increases (Dong et al., 2018). This suggests that while the VR‐HE can effectively trigger physiological arousal, it might not be intense enough to disrupt autonomic balance significantly.

Regarding RR, in both groups there was a noticeable increase in RR at follow‐up. Although RR is strongly related to stress (Nicolò et al., 2020), and similar findings were reported following exposure to both emotional and mental stressors (Hernando et al., 2015; Masaoka & Homma, 1997). However, given the fact that an increase in RR is not anticipated in the VBS condition, the increase reported could not be attributed solely to stress but also to its association with a higher workload, particularly since the measurements promptly followed cognitive testing (Charles & Nixon, 2019).

Despite changes in physiological parameters, participants did not report a corresponding change in subjective stress levels following the VR‐HE. This contrasts with previous research using the VR‐HE in civilian populations (Martens et al., 2019) and suggests that military personnel may have a higher threshold for reporting stress due to their training and experience (Chao et al., 2023). Moreover, this inconsistency between physiological and subjective stress responses observed in this study highlights the complexity of measuring stress. It suggests that physiological markers may provide more reliable indicators of stress than self‐reported measures, particularly in trained populations accustomed to high‐stress environments. Future research should continue to explore the relationship between physiological and subjective measures of stress to better understand how different populations perceive and report stress.

The lack of significant stress induction in the VBS condition aligns with Binsch et al. (2021), suggesting that VBS, even with its active, goal‐directed scenarios, may not yet be effective for stress‐related training in military contexts. Previous research has shown the efficacy of other combat games in eliciting stress responses in military populations (Bouchard et al., 2014; Porter & Goolkasian, 2019). Therefore, it appears that VBS scenarios may need enhancements in realism and interactivity to be effective stressors. The implications of these findings suggest a potential gap in current VBS design, necessitating further investigation into the elements that make VR environments stress‐inducing.

Regarding cognitive performance, no significant impairment was observed in either condition, contradicting the expected stress‐induced cognitive decline. This might be due to the modest stress response induced by the VR‐HE, seeing that mild stress does not necessarily impair the domains needed for these tasks (i.e. processing speed, selective attention, and response time) and can even enhance it (Qi et al., 2018; Shields et al., 2019). An alternative explanation for this outcome could be linked to the re‐test effect (Scharfen et al., 2018; Beglinger et al., 2005), where the repeated testing cancels out the decline in cognitive performance induced by the stressor.

### 4.1 Implications for stress research

The findings of this study have broader implications for stress research and training. First, the effectiveness of simulation‐based training tools in inducing stress needs to be carefully evaluated and optimised. The results indicate that simply enhancing the immersive experience may not be sufficient; scenarios must also be engaging and realistic to effectively induce a stress response. This aligns with the broader literature emphasising the importance of scenario design in virtual training environments (Bouchard et al., 2014; Lackey et al., 2016; Servotte et al., 2020). For military applications, this could involve more dynamic and interactive scenarios that adapt to the actions and decisions of the participants, providing a sense of consequence and unpredictability that mirrors real‐world stressors. Enhancing the physical and sensory feedback within the virtual environments, such as incorporating haptic feedback or simulating environmental conditions (e.g. heat, noise), could further increase the realism and stress‐inducing potential of the scenarios.

Beyond military training, the implications of this study extend to other high‐stress occupations such as emergency responders, medical personnel, and law enforcement. Building upon these findings, training tools can be designed and improved to simulate stress situations specific to these professions. For example, aiding individuals to develop stress resilience improves their stress management by controlling their stress response, and enables optimal performance under moderate/controlled stress levels. By providing a safe and controlled environment for stress exposure, simulation training can allow individuals to practice and refine their coping strategies without the risk and costs associated with real‐life stressors.

### 4.2 Limitations

Evidently, this study presents several notable limitations. Firstly, a substantial portion (80%) of the subjects had no prior experience with VBS. Despite the single training scenario, several subjects struggled to effectively navigate VBS during the actual scenarios, which could potentially lead to feelings of frustration rather than stress. Future investigations would benefit from a more comprehensive training phase to familiarize participants with VBS. Moreover, participant's experience with the situations encountered in VBS may vary in this study due to differences in their roles, prior training, and individual exposure to similar scenarios in the line of duty.

Secondly, the VR‐HE condition demanded more physically active engagement, involving activities such as standing, walking, and crouching. However, due to participants' unfamiliarity with their surroundings and concerns about potential falls while wearing the VR headset, their movement was restricted, failing to match the intensity of our walking baseline measurements.

Thus, it is not expected that the difference in physiological measurements was attributable to movement. Nonetheless, data examination did reveal changes in cardiac parameters after standing up. When humans stand up, gravitational forces cause a sudden descend of blood from the thorax into the abdominal cavity and limbs triggering immediate peripheral vasoconstriction, causing an increase in HR (Quinn et al., 2022). In the current dataset an increase in HR and a decrease in HRV was observed. While this effect was not anticipated beforehand, validation through the smaller‐scale experiment among colleagues demonstrated a similar trend, albeit slightly smaller. Thus, it is crucial to consider this factor when interpreting the study's outcomes, as it partially explains the observed differences in HR and HRV. However, it is possible that this difference is an overestimation, considering that the baseline measurement was taken immediately after the 2‐min baseline walking phase. This may have resulted in already elevated baseline values compared to if the participants would have been sitting.

Thirdly, excessive movement during the VR‐HE introduced considerable noise in the respiration data, necessitating the exclusion of T1, T2, and T3 for analyses of RR. Consequently, our analysis could only compare baseline and follow‐up measurements of RR. This limitation underscores the need for caution when drawing conclusions about the impact of the VR‐HE on respiration dynamics.

## 5 CONCLUSION

In conclusion, while the prospect of employing a fully controllable military simulator for stress inoculation appears promising and aligns with the demand for tailored stress training, the findings of the present study did not lend support to the use of VBS for such purposes. Given the demonstrated efficacy of the VR‐HE, the subsequent course of action may involve the development of more immersive, intricate, and challenging scenarios, integrating them into a VR framework. By combining the heightened immersion and realism achievable through VR with more engaging, intricate scenarios, VBS could potentially become more effective in eliciting a stress response. Presently, VBS retains its value as a tool for military training in various domains, including decision‐making, location scouting, and communication training.

## CONFLICT OF INTEREST STATEMENT

The authors have declared that they have no conflict of interest.

## Supporting information

Supporting Information S1

## Data Availability

The data that support the findings of this study are available from the corresponding author upon reasonable request.
