# Supplementary material for: Examining the applicability of virtual battle space for stress management training in military personnel—A validation study
Source: Stress Health. 2024 Sep 28;40(6):e3475. doi: 10.1002/smi.3475 (PMC11636437; doi:10.1002/smi.3475)
Supplement: Supplementary file 1 — Supporting Information S1 [file SMI-40-e3475-s001.docx]

**Supplementary Information**

*VBS scenarios*

In the first VBS scenario, participants were assigned to guard a bridge to secure entrance to the city centre. Their task was to observe the main road and surroundings without specific details, fostering a sense of uncertainty. They could, however, move freely within their area. Players were armed with a M4A5 assault rifle with an ELCAN optical scope. The simulation was situated in a lively town with many pedestrians, bypassing vehicles, wandering animals, and activity from fellow military troops. All of which were pre-programmed within the VBS software. During the first five minutes of the simulation, various suspicious activities occurred, like passing cars, a man with binoculars observing the bridge, people making phone calls, and a military helicopter flying by. Then, a sudden explosion occurred which caused the screen of the player to shake vigorously. Immediately following the explosion, two opponents fire multiple shots in the player’s direction from behind to buildings in the distance. The player’s guard squad engaged. During the ambush, a truck approached the bridge. If the unguarded truck stayed still for five seconds at the bridge, it would explode, ending the scenario.

In the second scenario, participants were virtually situated in the passenger seat of a military ambulance. Their briefing outlined an explosion involving a civilian vehicle, resulting in one injured person. The ambulance, part of a convoy, was pre-programmed to reach its destination within. During the two-minute drive the player traversed through countryside terrain encountering a herd of animals crossing the road and passing multiple cars. Approaching a small village, the simulation depicted vehicles and people, with signs of wreckage evident as several vehicles blazed on the roadside. Upon arriving at the destination, a sudden explosion ensued, emitting a piercing beep, and causing the screen to go black momentarily. As the player regained consciousness, military colleagues were injured, and gunfire targeted their direction. Two colleagues lay wounded while the participant regained control of their avatar. Prompted at the screen’s top was the text: “You are under fire. Two heavily injured individuals lie on the ground. You are unarmed. Act swiftly”. The participant was, again, free to move around. The scenario concluded upon successfully guiding the injured colleagues to safety or when seven minutes elapsed.

**Supplementary Table 1.** A summary of the averaged outcomes of all parameters across the experimental time course.

|  | Mean ± SD | | | | | |
| --- | --- | --- | --- | --- | --- | --- |
|  | T0 (baseline) | T1 | T2 | T3 | T4 | N |
| HR (bpm) |  |  |  |  |  |  |
| VBS | 69.53 ± 9.41 | 67.25 ± 10.48 | 68.90 ± 9.80 | 68.74 ± 9.26 | 68.24 ± 10.30 | 18 |
| VR-HE | 69.53 ± 14.17 | 89.80 ± 13.76 | 87.75 ± 12.26 | 81.10 ± 13.37 | 68.80 ± 9.16 | 18 |
| HRV (ms) |  |  |  |  |  |  |
| VBS | 49.94 ± 20.77 | 48.08 ± 24.64 | 48.39 ± 23.04 | 46.74 ± 21.05 | 43.62 ± 21.51 | 18 |
| VR-HE | 51.08 ± 26.86 | 29.71 ± 15.92 | 40.75 ± 23.40 | 40.10 ± 19.89 | 43.62 ± 21.51 | 18 |
| RR (bpm) |  |  |  |  |  |  |
| VBS | 12.90 ± 3.45 | - | - | - | 17.38 ± 2.79 | 20 |
| VR-HE | 13.58 ± 3.49 | - | - | - | 16.99 ± 3.28 | 20 |
| STAI score |  |  |  |  |  |  |
| VBS | 65.30 ± 14.49 | - | - | - | 64.85 ± 14.68 | 20 |
| VR-HE | 62.30 ± 14.32 | - | - | - | 62.05 ± 15.07 | 20 |
| VAS score |  |  |  |  |  |  |
| VBS | 2.25 ± 2.31 | - | - | - | 2.05 ± 2.19 | 20 |
| VR-HE | 2.10 ± 2.29 | - | - | - | 1.95 ± 1.93 | 20 |
| PVT (ms) |  |  |  |  |  |  |
| VBS | 346.10 ± 23.59 | - | - | - | 361.09 ± 35.46 | 20 |
| VR-HE | 349.34 ± 22.45 | - | - | - | 350.47 ± 32.79 | 20 |
| Stroop effect (ms) |  |  |  |  |  |  |
| VBS | 69.04 ± 56.26 | - | - | - | 39.31 ± 63.18 | 20 |
| VR-HE | 64.77 ± 62.14 | - | - | - | 43.94 ± 80.62 | 20 |

**Supplementary Table 2.** A summary of the main effects of repeated measures ANOVAs on stress parameters and cognitive performance. FDR adjusted *p*-values that are no longer significant are shown in bold.

|  | F | *df* | *df error* | Unadjusted *p*-value | FDR adjusted *p*-value |
| --- | --- | --- | --- | --- | --- |
| **Heart Rate** |  |  |  |  |  |
| Time | 32.465 | 2.678 | 45.519 | 0.00001 | <0.001 |
| Condition | 32.606 | 1 | 17 | 0.00001 | <0.001 |
| Time x Condition | 53.368 | 2.565 | 43.611 | 0.00001 | <0.001 |
| **Heart Rate Variability** |  |  |  |  |  |
| Time | 5.059 | 4 | 68 | 0.001 | 0.0042 |
| Condition | 4.402 | 1 | 17 | 0.051 | 0.113 |
| Time x Condition | 4.330 | 4 | 68 | 0.004 | 0.014 |
| **Respiration Rate** |  |  |  |  |  |
| Time | 51.083 | 2.666 | 50.657 | 0.00001 | <0.001 |
| Condition | 18.764 | 1 | 19 | 0.667 | 0.870 |
| Time x Condition | 5.221 | 4 | 76 | 0.094 | 0.180 |
| **Visual Analogue Scale** |  |  |  |  |  |
| Time | 0.921 | 1 | 19 | 0.807 | 0.922 |
| Condition | 0.748 | 1 | 19 | 0.12 | 0.210 |
| Time x Condition | 0.045 | 1 | 19 | 0.93 | 0.977 |
| **State-Trait Anxiety Inventory** |  |  |  |  |  |
| Time | 0.062 | 1 | 19 | 0.349 | 0.564 |
| Condition | 2.648 | 1 | 19 | 0.398 | 0.570 |
| Time x Condition | 0.008 | 1 | 19 | 0.834 | 0.922 |
| **Psychomotor Vigilance Task** |  |  |  |  |  |
| Time | 4.201 | 1 | 19 | 0.054 | 0.113 |
| Condition | 0.718 | 1 | 19 | 0.407 | 0.570 |
| Time x Condition | 4.595 | 1 | 19 | 0.045 | **0.113** |
| **Stroop Task** |  |  |  |  |  |
| Time | 6.048 | 1 | 19 | 0.024 | **0.072** |
| Condition | 0.000 | 1 | 19 | 0.987 | 0.987 |
| Time x Condition | 0.149 | 1 | 19 | 0.704 | 0.870 |
